# Ultrasound-guided versus fluoroscopy-guided lumbar selective nerve root block: a retrospective comparative study

**DOI:** 10.1038/s41598-024-53809-3

**Published:** 2024-02-08

**Authors:** Bowen Wang, Yang Sun, Jitao Zhang, Hailan Meng, Hong Zhang, Lequn Shan

**Affiliations:** 1 Yan’an University, Yan’an, 716000 Shannxi China; 2 The Ultrasound Department of Honghui Hospital, Xi’an Jiaotong University, Xi’an, 710054 Shannxi China; 3 The Spine Surgery Department of Honghui Hospital, Xi’an Jiaotong University, Xi’an, 710054 Shannxi China

**Keywords:** Ultrasound, Fluoroscopy, Selective nerve root block, Lumbar radiculopathy, Diseases, Clinical trial design

## Abstract

The purpose of this study is to compare the accuracy and effectiveness of ultrasound-guided and fluoroscopy-guided lumbar selective nerve root block (SNRB), and to explore the feasibility of ultrasound-guided methods. This retrospective study included patients with lumbar radicular pain who underwent ultrasound-guided and fluoroscopy-guided selective nerve root block at Honghui Hospital Affiliated to Xi’an Jiaotong University from August 2020 to August 2022. Patients were divided into U-SNRB group and F-SNRB group according to ultrasound-guided or fluoroscopy-guided selective nerve root block. There were 43 patients in U-SNRB group and 20 patients in F-SNRB group. The pain visual analogue scale (VAS) scores, Japanese Orthopaedic Association (JOA) scores, related indexes and complications were recorded and compared between the two groups before, 30 min, 1 month and 6 months after block. To evaluate the feasibility, accuracy and effectiveness of ultrasound-guided selective nerve root block. There were no complications in the process of selective nerve root block in both groups. The operating time and the times of closing needle angle adjustment in U-SNRB group were better than those in F-SNRB group, and the difference was statistically significant (P < 0.05). The VAS score and JOA score of patients in the two groups were significantly improved 30 min after block, 1 month and 6 months after block, and the difference was statistically significant (P < 0.05). There was no significant difference between the two groups (P > 0.05). The accuracy of ultrasound-guided selective nerve root block and the degree of pain relief of patients were similar to those of fluoroscopy guidance, but the operation time and needle angle adjustment times were significantly less than that of fluoroscopy, and could effectively reduce radiation exposure. Therefore, it can be used as a better way to guide for choice.

## Introduction

Lumbar nerve root pain refers to pain caused by specific nerves of the lumbar spine that can radiate from the lower back to the legs or beyond. Patients often have mechanical lesions of the spine, such as intervertebral disc herniation or spinal canal stenosis caused by structural hyperplasia of ligamentum flavum and articular process^1^. Its clinical manifestations are usually complex, and the symptoms of some patients are not consistent with the imaging examination. Because the relief of pain is closely related to the effectiveness of treatment and the subjective feelings of patients, it has gradually attracted more and more clinicians' attention in recent years. However, a simple combination of symptoms and imaging examinations is often considered insufficient to make a diagnosis^2^. It is generally believed that selective nerve root block is of great significance for the diagnosis and treatment of the involved segments. Selective nerve root block often needs to be done under the guidance of X-ray or ct to ensure accuracy and avoid complications. In contrast, ultrasound, as a relatively new alternative technology, has portable equipment and low price, and can minimize the radiation exposure of patients. Ultrasound equipment can display tip track, injection site and drug diffusion in real time, and it has been proved that it can be used in the guidance of cervical nerve root block^3^. The success of lumbar selective nerve root block depends on the accuracy of the injection site and the relief of patients' corresponding clinical symptoms. The purpose of this study was to evaluate the effectiveness and accuracy of ultrasound-guided lumbar selective nerve root block, compared with fluoroscopy-guided injection.

## Materials and methods

This study is a retrospective comparative study of previous medical records. Patient privacy and data confidentiality were maintained throughout the study.Ethics Committee of the Red Society Hospital Affiliated to Xi'an Jiaotong University approved the study. All experiments were performed in accordance with relevant guidelines and regulations.

### Inclusion and exclusion criteria

#### Inclusion criteria

(1) Patients with complete data underwent selective nerve root block under the guidance of ultrasound or fluoroscopy. (2) Patients with nerve root low back pain diagnosed by clinical manifestation, physical examination, electromyography and imaging examination. (3) The patients who received nerve root closure for more than half a year and had complete follow-up data.

#### Exclusion criteria

(1) Conservative treatment or surgery, unclosed patients. (2) Lack of clinical data. No obvious nerve root symptoms were found in clinical manifestations. (3) Local or systemic infection, or patients with irreversible coagulation disorders. (4) Patients with allergies to corticosteroid drugs or anesthetics. (5) Pregnancy.

Combined with clinical symptoms, physical examination and imaging examination. Select the segments that may be involved and mark them on the skin, nerve root blocking injection was carried out in the corresponding segments. Statistics of clinical data: VAS, JOA score of patients, record the operation time. Record demographic data.

### Ultrasound-guided nerve root block process

**Figure 1:**
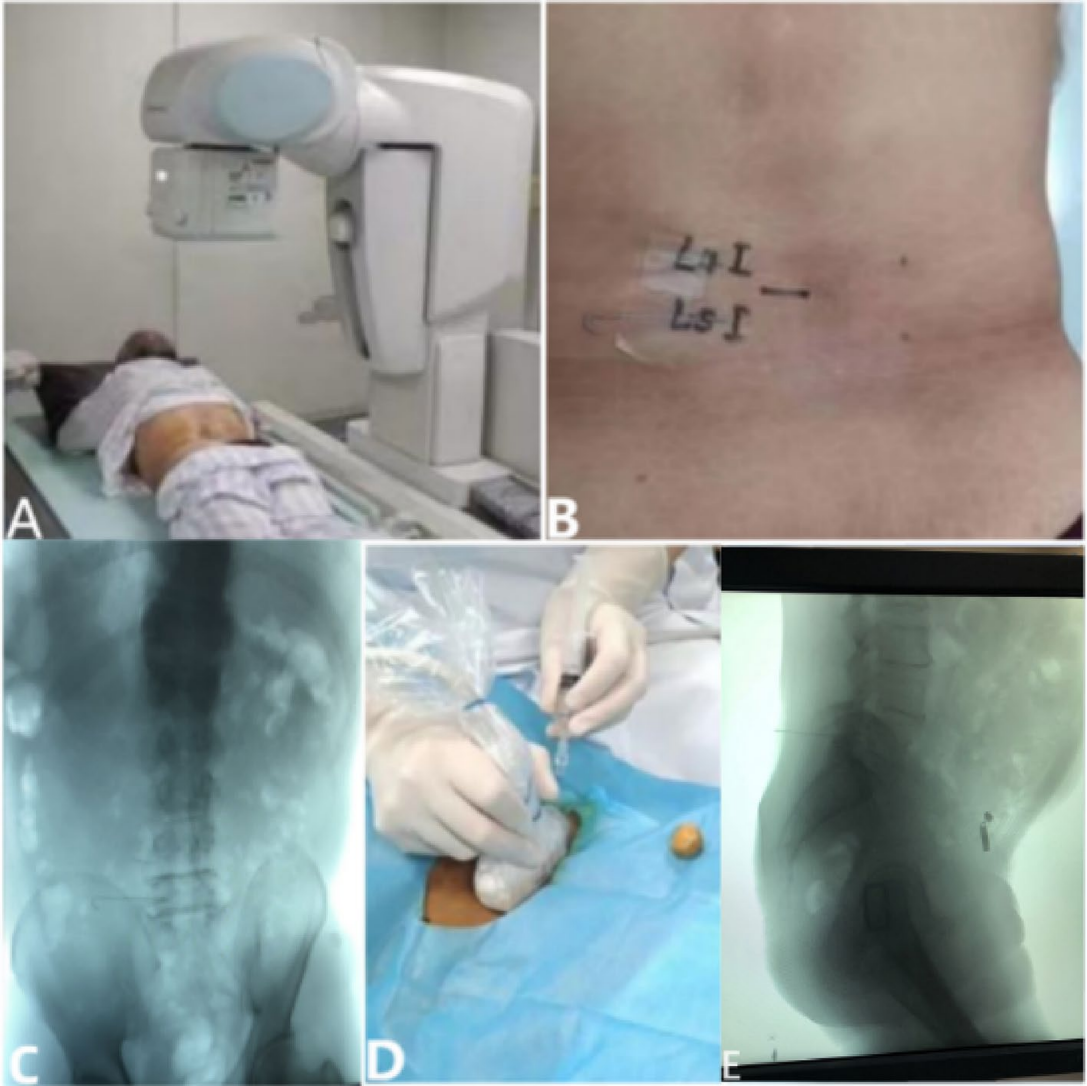
X-ray verification picture: (**A**) the ultrasound equipment was placed in the X-ray examination room. The patient lies prone on the X-ray bed and the clothes are pulled up to expose the waist and back. (**B**) The planned block segments are marked on the body surface, and an X-ray marker is attached. (**C**) X-ray shows that the marker is in the correct position. (**D**) The block needle was placed under the guidance of ultrasound. (**E**) X-ray verification: the block needle is accurately in place.

The patient is in a prone position with his head tilted to one side. Start recording the time. The operation was carried out by an ultrasound doctor with rich experience in ultrasound needle placement. The operation was performed using standard ultrasound equipment (GE LOGIQ E9), using a probe: convex array probe C1-6. Cover the ultrasonic transducer with aseptic packaging. Sterile ultrasonic gel is applied to the marked skin. Ultrasound doctors start from the sacral vertebra, identify the vertebral body segment by segment along the spinal segment, and judge whether the bony structure of the vertebral body can be accurately identified when it reaches the required injection segment. When the ultrasound transducer reaches the corresponding spinal segment and can accurately identify the bone structures of the vertebral body, such as spinous processes, transverse processes, and facet joints, we consider the ultrasound imaging to be clear, and then start the needle placement procedure. A spinal puncture needle (20G puncture needle) is accurately inserted into the skin, and the angle and position are determined by the transducer and anatomy. Our target is the back of the intervertebral foramen and the needle is inserted along the lateral edge of the lamina, where the beating of the nerve root can be seen. In order to evaluate the accuracy of the tip, the tip was verified by X-ray fluoroscopy(Fig. 1). If the tip of the needle deviates or cannot reach the target, change the angle or return the needle to re-select the entry point under the guidance of ultrasound. If the tip of the needle is placed correctly, record the time taken during the operation. Injection (2% lidocaine 1 ml + 0.9% sodium chloride injection 1 ml + compound betamethasone injection 1 ml (4 mg), total 3 ml) mixture.

### Fluoroscopy-guided nerve root block process

The position of the patient is the same as that of ultrasound. An opaque development mark was placed on the body surface to predict the spinal segments and needle tracks. Radiography was used to obtain the anterior and lateral images of the lumbar spine, combined with the placement of markers to accurately select the needle track. Select the appropriate needle insertion angle and mark the needle insertion position, combined with the image to measure the needle insertion depth. The operation was performed by a spinal surgeon with many years of fluoroscopic-guided puncture experience. Complete sterilization, towel laying and other aseptic operations. After local anesthesia, the puncture needle was placed according to the original angle and depth. Take pictures of the anterior and lateral position of the lumbar vertebrae again or more times to determine the position of the needle tip until it is correctly positioned. The type of drug injected is consistent with the dose and ultrasound. Record the time used. Ask the patient about the degree of pain relief and assist in sorting out the clothes.

### Clinical data review

The VAS and JOA scores and the remission rate (VAS < 3) were collected before and 30 min after injection, 1 month and 6 months after injection. The accuracy of ultrasonic examination has been verified by X-ray.

There is no gold standard for the diagnosis of lumbar nerve root pain, on the basis of patient history and clinical examination, combined with imaging examination and pain relief after nerve root block, neuroelectrophysiological examination is also an important diagnostic method^4,5^.

### Statistical analysis of data

Statistical analysis was carried out by using SPSS26.0 software (IBM, USA).The continuous measurement data were expressed by mean ± standard deviation (x ± s).Shapiro–Wilk normality test was carried out on the observed index data of the two groups. Paired t test was used to compare the data before and after block in the same group in accordance with normal distribution, and Wilcoxon symbolic rank sum test was used for data of non-normal distribution. Comparison of data between two groups using two independent sample t-test, and Mann-Whitney U test was used to test the data of non-normal distribution. The count data were expressed as rates and were tested by χ^2^ test or Fisher test. The test level α = 0.05, P < 0.05 was statistically significant.

### Institutional review board statement

Ethics Committee of the Red Society Hospital Affiliated to Xi'an Jiaotong University approved the study. Ethical review number: 202304003.

### Informed consent statement

The Red Society Hospital affiliated to Xi’an Jiaotong University approved the waiver of informed consent.

## Result

122 lumbar segments injected by 63 patients were collected retrospectively. The process of nerve root block was smooth in both groups, and there was no nerve root injury. In U-SNRB group, there were 11 cases of unilateral single segment (1 case of L1/2, 1 case of L2/3, 1 case of L3/4, 4 cases of L4/5, 4 cases of L5/S1), 2 cases of unilateral and double segments (L4/5, L5/S1), 26 cases of bilateral single segment (2 cases of T12/L1, 2 cases of L1/2, 1 case of L2/3, 3 cases of L3/4, 14 cases of L4/5, 4 cases of L5/S1), 3 cases of bilateral double segment (2 cases of L3/4 and L4/5, 1 case of L2/3 and L3/4), 1 case of bilateral three segments (L2/3, L4/5 and L5/S1). In F-SNRB group: 7 cases of unilateral and single segment (1 case of L1/2, 2 cases of L2/3, 2 cases of L3/4, 2 cases of L4/5), 1 case of unilateral double segment (L4/5 and L5/S1), 10 cases of bilateral single segment (2 cases of T12/L1, 1 case of L1/2, 3 case of L3/4, 4 case of L4/5), 2 cases of bilateral double segment (1 case of L3/4 and L4/5, 1 case of L4/5 and L5/S1).

The average age of patients in ultrasound group and perspective group was 63 ± 9 years old and 67 ± 9 years old respectively, and there was no significant difference between the two groups (P > 0.05).In addition, there was no significant difference in sex, height, weight, BMI and blocked nerve root segment between the two groups (Table 1).

**Table 1:** General characteristics of patients.

Index	F-SNRB	U-SNRB	P value
Total	20	43	–
Male/female	10/10	14/29	0.185
Age	67 ± 9	63 ± 9	0.134
Height/cm	166 ± 9	164 ± 7	0.268
Weight/kg	67 ± 10	65 ± 10	0.616
BMI	24.20 ± 2.58	24.31 ± 3.31	0.893
Block segment of lumbar vertebra			–
T12, L1	2 (8.70%)	2 (4.00%)	
L1, 2	2 (8.70%)	3 (6.00%)	
L2, 3	2 (8.70%)	4 (8.00%)	
L3, 4	6 (26.09%)	7 (14.00%)	
L4, 5	9 (39.13%)	23 (46.00%)	
L5, S1	2 (8.70%)	11 (22.00%)	

Notes: The continuous measurement data were expressed by mean ± standard deviation (x ± s).The count data were expressed as rates.

F-SNRB, fluoroscopy-guided lumbar selective nerve root block. U-SNRB, ultrasound-guided lumbar selective nerve root block. BMI, body mass index.

Intra-group comparison between two groups both showed satisfactory clinical effects (P < 0.05). In the F-SNRB group, pain relief was achieved in 15 patients (VAS < 3) 30 min after injection, and in 19 patients (95%) at 1 month after injection (VAS < 3).In U-SNRB group, pain was relieved in 33 patients (VAS < 3) 30 min after injection, and in all patients (VAS < 3) 1 month after injection (Table 2).

**Table 2:** Blocking effect.

	Before block	30 min	1 month	6 months
VAS				
U-SNRB	6.7 ± 1.2	2.1 ± 0.7*	1.8 ± 0.4*	2.2 ± 0.6*
F-SNRB	6.5 ± 1.4	2.2 ± 0.7*	1.9 ± 0.4*	2.2 ± 0.6*
JOA				
U-SNRB	15.1 ± 2.4	20.9 ± 1.7*	22.8 ± 1.3*	23.1 ± 1.1*
F-SNRB	15.6 ± 2.8	19.4 ± 1.2*	23.2 ± 1.5*	24.4 ± 1.1*

Notes: The continuous measurement data were expressed by mean ± standard deviation (x ± s).

VAS scores, visual analogue scale scores. JOA scores, Japanese Orthopaedic Association scores. F-SNRB, fluoroscopy-guided lumbar selective nerve root block. U-SNRB, ultrasound-guided lumbar selective nerve root block.

*P < 0.05: compared with those before block, the scores of patients at each time point were significantly different.

The number of needle angle adjustment and operation time in U-SNRB group were significantly less than those in F-SNRB group, and there are significant differences (P < 0.05) (Table 3).

**Table 3:** Main research indicators.

Index	Fluoroscopic	Ultrasound	P value
Needle angle adjustment/time	4 ± 1	1 ± 1	P < 0.05
Operating time/min	18 ± 2	9 ± 2	P < 0.05
Radiation exposure	depending on the number of fluoroscopic	None	–

Notes: The continuous measurement data were expressed by mean ± standard deviation (x ± s).

The amount of X-ray radiation is determined by the number of fluoroscopy, and the ultrasonic equipment has no ionizing radiation.

No complications were reported in all patients.

## Discussion

Lumbar radiculopathy is a common disease of the spine, usually caused by mechanical compression or local inflammation to stimulate the nerve root. It is estimated that the prevalence rate is 3–5% of the population^6^.Clinically, routine treatment includes conservative treatment and surgical treatment, and surgical treatment is usually carried out when conservative treatment is ineffective. However, it has been reported in the literature that 5–36% of patients are at risk of failure of surgical treatment, and low back pain and leg pain will occur again within 2 years after operation^7^. The main reason is that the diagnosis of responsibility segment is not clear. A clear diagnosis is very important for the choice of treatment and surgical segments. However, when the clinical manifestations of the patients are not consistent with the imaging results, or when the patients are suspected of multi-segmental radiculopathy, the routine diagnosis cannot be completely accurate^8^. Additional diagnostic methods are usually required. As the “gold standard” of diagnosis, SNRB can judge the possible segment of responsibility according to the degree of pain relief after injection^9,10^. However, some researchers have reported the emergence of false positive rate, which is due to the accuracy of puncture and doubt whether the solution has reached the target point^11,12^. In order to improve the accuracy of block puncture and reduce complications, X-ray fluoroscopy or ct guidance is often used in clinic. It has been proved that under the two guidance methods, the accuracy of lumbar nerve root block can reach more than 90%^13–15^. But at the same time, the disadvantages of the two methods have been paid more and more attention by clinicians, such as longer operating time, inevitable risk of radiation exposure and so on^16^.

**Figure 2:**
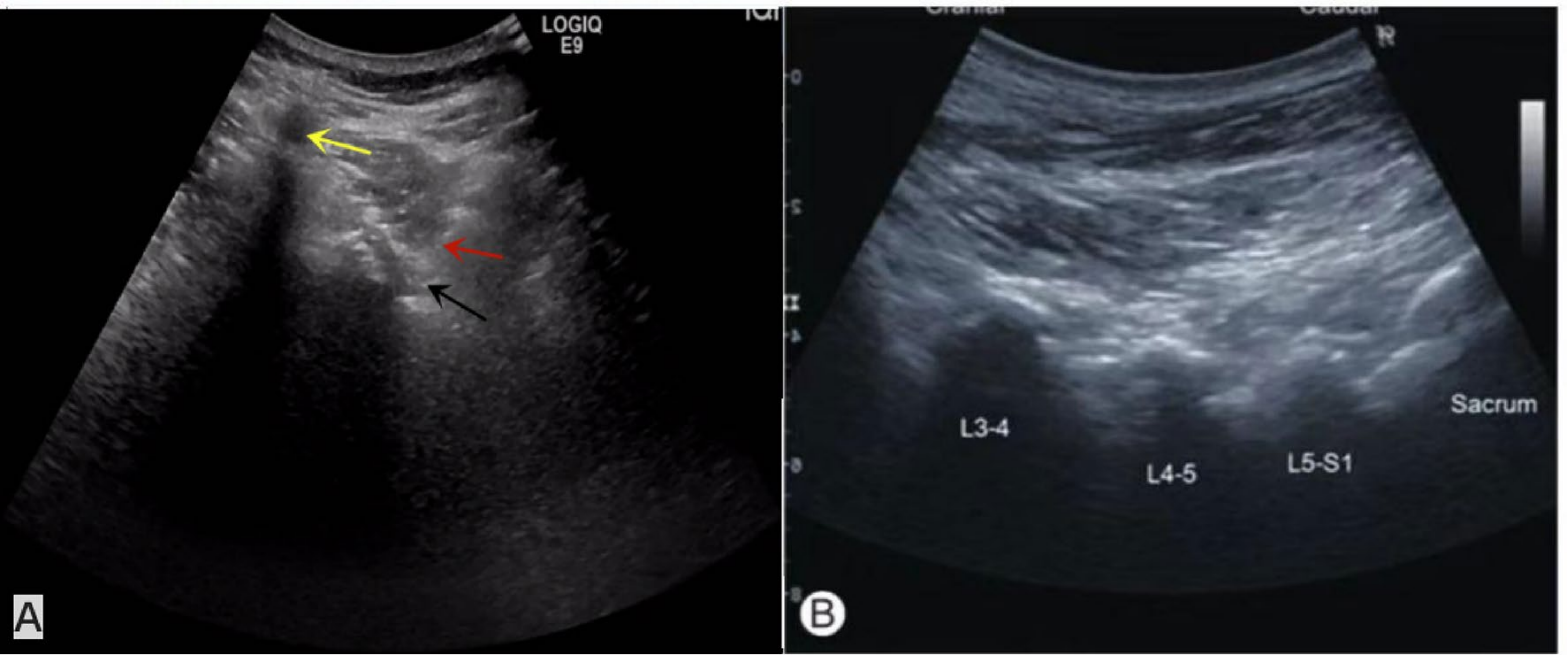
Ultrasonic location picture: (**A**) The yellow arrow shows the spinous process. The red arrow shows the tip of the needle. The black arrow shows the nerve root. (**B**) The long axis of the ultrasonic probe is placed parallel to the long axis of the body, and the lumbar segments are identified by the sacrum in turn.

In this retrospective study, the clinical results of ultrasound-guided SNRB and fluoroscopy-guided SNRB were compared to prove the accuracy of ultrasound-guided mode. The results showed that 30 min after the completion of block injection, the pain relief of patients in ultrasound guidance group and fluoroscopy guidance group was 75% and 77% respectively, and there was no significant difference between the two groups. At 1 and 6 months after injection, there was no significant difference between ultrasound guidance and fluoroscopy guidance. In this study, patients with lumbar nerve root pain were followed up for 6 months, there was no significant difference between ultrasound guidance and fluoroscopy guidance in pain control and functional improvement. However, compared with fluoroscopy guidance, the operation time and needle angle adjustment times of SNRB guided by ultrasound were significantly less than those guided by fluoroscopy. We believe that the higher efficiency of ultrasound guidance comes from its unique advantages, such as real-time, can clearly display blood vessels, nerves, soft tissue and so on (Fig. 2). With the help of ultrasonic probe, ultrasound doctors preset the needle position and puncture angle in advance, and can monitor the movement of puncture needle in real time, so as to effectively avoid the occurrence of puncture complications. Previous reports of some complications, such as nerve injury, allergic reactions to local anesthetics, arteriole occlusion and tissue injury^17^. The number of selected cases in this study is small, there are no complications, and the results are limited, so further and larger sample size studies are needed to determine the degree of complications related to the SNRB procedure. In addition, for accuracy, repeated fluoroscopy makes patients and doctors inevitably suffer from the harm of ionizing radiation, ultrasound equipment can effectively avoid.

In previous studies, ultrasound-guided SNRB has been reported. Kim et al.^18^ can quickly and accurately identify nerve roots by identifying the transverse process and facet joint structure of lumbar joints, so as to guide the block process and improve the accuracy. Kim et al.^2^ described and compared two kinds of needle approach: nerve root blocking paramedian sagittal approach and paramedian sagittal oblique approach. It is proved that it is easier for drugs to enter the intervertebral foramen by paracentric sagittal oblique approach than by paramittal sagittal approach, and can more significantly reduce the pain. In this study, we placed the ultrasonic probe parallel to the long axis of the body, identified the vertebrae one by one from the bottom to the top of the sacral vertebrae, and rotated the probe against the skin 90 degrees when we reached the required injection segments, to judge and identify the spinous process, transverse process and facet joint structure of the lumbar joint, so that the block effect can be achieved accurately. In the past, some studies have compared ultrasound-guided and fluoroscopic-guided lumbar facet joint closure and cervical selective nerve root closure. In contrast, the accuracy and clinical effect of ultrasound are satisfactory^19–21^. This study creatively describes and proves the accuracy and effectiveness of ultrasound guidance by comparing with fluoroscopic guidance.

## Limitations

(1) Although we have established inclusion and exclusion criteria, there is still heterogeneity in patient selection, and we are unable to confirm whether patients follow the doctor’s advice and will receive other treatments during treatment and follow-up. (2) The treatment process is carried out by a single ultrasound doctor, there may be deviations in the clinical understanding of spinal diseases, and the results can only reflect the experience of one doctor, which limits the generalization of the results to a certain extent. Larger sample size and more universal research are needed in the future.

## Conclusion

The accuracy and pain relief degree of ultrasound guidance were similar to those of fluoroscopy guidance, but the operation time and needle angle adjustment times were significantly less than that of fluoroscopy guidance. The ultrasonic equipment can guide the puncture needle in real time, reduce the radiation exposure of operators and subjects, and is more efficient, safe and cost-effective. It can be used as a better choice for clinical diagnosis and treatment.

## Data Availability

All relevant data were provided in the manuscript.
