# Author Correction: Ultrasound-guided versus fluoroscopy-guided lumbar selective nerve root block: a retrospective comparative study

**DOI:** 10.1038/s41598-024-59217-x

**Published:** 2024-04-11

**Authors:** Bowen Wang, Yang Sun, Jitao Zhang, Hailan Meng, Hong Zhang, Lequn Shan

**Affiliations:** 1 Yan’an University, Yan’an, 716000 Shannxi China; 2 The Ultrasound Department of Honghui Hospital, Xi’an Jiaotong University, Xi’an, 710054 Shannxi China; 3 The Spine Surgery Department of Honghui Hospital, Xi’an Jiaotong University, Xi’an, 710054 Shannxi China

Correction to: *Scientific Reports* 10.1038/s41598-024-53809-3, published online 08 February 2024

The original version of this Article omitted an affiliation for Bowen Wang. The correct affiliations are listed below.

Yan’an University, Yan’an 716000, Shannxi, China.

The Spine Surgery Department of Honghui Hospital, Xi’an Jiaotong University, Xi’an 710054, Shannxi, China.

Yang Sun, Jitao Zhang and Hailan Meng were incorrectly affiliated with ‘The Ultrasound Department of Honghui Hospital, Xi’an Jiaotong University, Xi’an 710054, Shannxi, China.’ The correct affiliation is listed below.

The Spine Surgery Department of Honghui Hospital, Xi’an Jiaotong University, Xi’an 710054, Shannxi, China.

In addition, Affiliation 2 was incorrectly given as ‘The Ultrasound Department of the Red Society Hospital Affiliated to Xi’an Jiaotong University, Xi’an 710054, Shannxi, China.’ The correct affiliation is listed below.

The Ultrasound Medical Center of Honghui Hospital, Xi’an Jiaotong University, Xi’an 710054, Shannxi, China.

Affiliation 3 was incorrectly given as ‘The Spine Surgery Department of the Red Society Hospital Affiliated to Xi’an Jiaotong University, Xi’an 710054, Shannxi, China.’ The correct affiliation is listed below.

The Spine Surgery Department of Honghui Hospital, Xi’an Jiaotong University, Xi’an 710054, Shannxi, China.

The original Article has been corrected.

